# Measurement of special access to home visit nursing services among Japanese disabled elderly people: using GIS and claim data

**DOI:** 10.1186/s12913-017-2322-0

**Published:** 2017-05-30

**Authors:** Takashi Naruse, Hiroshige Matsumoto, Mahiro Fujisaki-Sakai, Satoko Nagata

**Affiliations:** Department of Community Health nursing, Graduate School of Medicine, The University of Tokyo, 7-3-1, Hongo, Bunkyo-ku, Tokyo, 113-0033 Japan

**Keywords:** Home visit nursing, Allocation, Service use, Geographic information systems (GIS)

## Abstract

**Background:**

Home care service demands are increasing in Japan; this necessitates improved service allocation. This study examined the relationship between home visit nursing (HVN) service use and the proportion of elderly people living within 10 min’ travel of HVN agencies.

**Methods:**

The population of elderly people living within reach of HVN agencies for each of 17 municipalities in one low-density prefecture was calculated using public data and geographic information systems.

Multilevel logistic analysis for 2641 elderly people was conducted using medical and long-term care insurance claims data from October 2010 to examine the association between the proportion of elderly people reachable by HVNs and service usage in 13 municipalities. Municipality variables included HVN agency allocation appropriateness. Individual variables included HVN usage and demographic variables.

**Results:**

The reachable proportion of the elderly population ranged from 0.0 to 90.2% in the examined municipalities. The reachable proportion of the elderly population was significantly positively correlated with HVN use (odds ratio: 1.938; confidence interval: 1.265–2.967).

**Conclusions:**

Residents living in municipalities with a lower reachable proportion of the elderly population are less likely to use HVN services. Public health interventions should increase the reachable proportion of the elderly population in order to improve HVN service use.

## Background

In 2009–2010, 28 Organization for Economic Co-operation and Development countries ranked ensuring and improving the quality of long-term care services as the second most important policy priority [1]. In Japan, public health professionals in local municipalities are responsible for improving long-term care systems for disabled elderly [2]. The equitable provision of accessible services is among the most important aspects of municipalities’ long-term care system.

Public health professions are also required to assess and improve regional resources accessible to long-term care services [2]. ‘Access’ includes many aspects of services. Public health professions must consider a range of aspects of accessibility when assessing and improving their municipality’s long-term care system; however, it remains difficult to assess long-term care services’ geographic accessibility.

In Japanese municipalities, long-term care services are concentrated in urban areas due to greater market opportunities [3]. This suggests that availability is lower in rural areas; however, municipality residents can obtain long-term care services from outside of their municipality. Hence, a smaller number of service providers within a given municipality does not necessarily indicate poor availability. Geographical distribution of resources, as well as resource volume (number of resources relative to the population), must be included in an adequate explanation of service availability when resources are not homogeneously geographically distributed [4]. Elucidatory measurement of municipal long-term care services’ geographic accessibility will facilitate long-term care system planning.

In this study, the travel time from residents’ homes to long-term care service locations was measured as an index of geographic accessibility. Extensive research examining healthcare allocation has confirmed the effect of service accessibility on health outcomes [5–7]. Geographic accessibility reflects the number of potential service users living near service locations [7]. Geographic accessibility is positively correlated with residents’ health. Previous research has measured services’ geographic accessibility by using geographic information systems (GIS) to analyse traffic networks. GISs can determine geographic accessibility from the locations of service users’ residences and service providers. For example, Todd et al. measured the population reachable from nearby general practice and community pharmacy services and converted this information into area specific index values of geographic accessibility [7].

This research examined home visiting nursing (HVN) services in Japan. HVNs are among the most widely used home-based, long-term care services for disabled elderly people [8]. HVN service usage reduces hospital and nursing home admissions and hospital stay duration [9]. Additionally, HVN service usage is covered by long-term care and health care insurance and is easily accessible to disabled elderly people. Physicians direct service commencement; professional care managers organize almost all visits. Nurses employed by an HVN agency typically provide HVN services. The number of HVN agencies has increased as society has aged; in 2014, approximately 7903 agencies existed in 1731 municipalities across 47 prefectures of Japan [10].

Health and long-term care insurance covering HVN is established and the number of HVN agencies has grown; however, unmet HVN service needs exist [11–13]. Examining factors affecting service usage may help to address unmet service needs. Numerous studies have reported individual factors correlated with service use [14–18]; measurement of these factors may help public health nurses to detect populations likely to have unmet HVN needs. Regarding community-level factors, socioeconomic status [19], poverty and crime, deterioration of trust, community relationships, and resources affect nurses’ home visitation for maternal heath purposes [20]; however, previous research has not examined HVN agency allocation’s relationship with HVN service usage. This preliminary study aimed to develop an index of HVN agency accessibility that may inform judgements of the appropriateness of service allocation within municipalities.

## Methods

We calculated accessibility indexes for each examined municipality and then compared HVN agency allocation with HVN service use among disabled elderly people. We hypothesized that more aged and disabled people would use HVN services in municipalities with higher accessibility index values. Insurance claim data was used to test this hypothesis. Insurance claim data can indicate every disabled elderlies’ medical service use (e.g., physician examination, HVN and admission) and ling-term care service use (e.g., home visit services including HVN, day care, and institutional services).

**Fig. 1:**
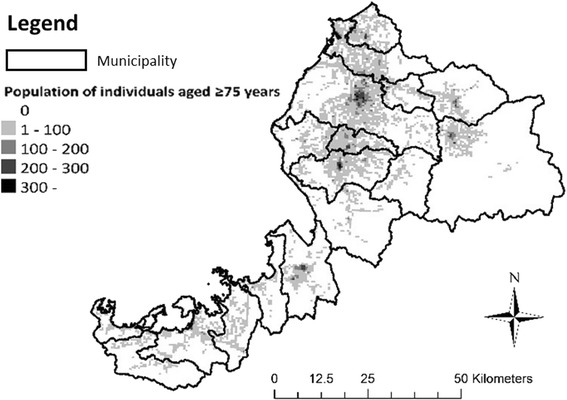
Population density in A Prefecture

This study analysed the following data types: publicly available data on the elderly population and HVN agency addresses, and long-term and health care insurance claim data for claims on HVN services. Municipalities’ HVN agency allocation characteristics were calculated using the former data type; elderly people’s HVN service usage and municipalities’ service agency allocation characteristics were examined using the latter data type. This study was conducted as part of a joint research project in the Institute of Gerontology Study. We received written consent to use insurance claim data in an anonymous electronic format from the A Prefecture National Health Insurance Federation and Association of medical care services for older senior citizens. A prefecture is one of Japan’s 47 prefectures. It includes 17 municipalities. Prefectures form the second level of jurisdiction and administrative division of Japan. Its population density is less than the national average, and the population does not distribute homogeneously (Fig. 1). Medical and long-term care service agencies (e.g., HVN agencies and home care clinics) tended to be on higher population density areas. This population density and distribution, however, is common among other Japanese prefectures, with the exception of the salient metropolitan areas like Tokyo and Osaka. This federation insures all 17 municipalities of A prefecture and manages claims data. Insured individuals were informed of the research through a public relations paper issued by A prefecture. Among the 17 municipalities studied, there were no differences in HVN system and insurance coverage. All clients were required to-payment for receiving HVN services. If a client could not pay, Japanese welfare system can support co-payment in order to prevent unmet HVN needs among all disabled elderlies.

The ethics committee of the Graduate School of Medicine at the [blinded for review] committee approved this study.

### Municipality variables

Municipalities’ HVN agency allocation appropriateness was measured by calculating the proportion of elderly people who lived near HVN agencies in each municipality. Proportions ranged from 0.0% (no elderly people in the municipality lived near an agency) to 100.0% (all elderly people in the municipality lived near an agency).

**Fig. 2:**
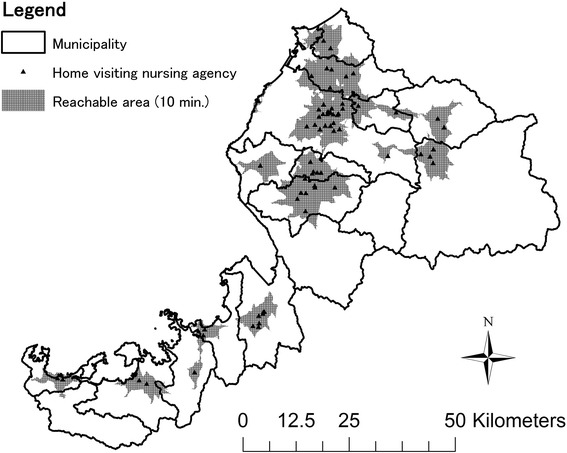
Home visiting nursing agencies’ reachable areas in A Prefecture

We divided each municipality into 0.5-square-kilometre areas (termed ‘meshes’), each of which was associated with the number of people who lived in the area aged ≥75 years (data obtained from the 2010 Japan National Population Survey) [21]. We subsequently located all HVN agencies on the divided map, and established the agencies’ ‘reachable area’ using the Arc GIS geographic information system program (Esri, Redlands, CA, USA; Fig. 2). Agencies’ reachable areas covered the geographical area ≤ 10 min’ travel from the agency by car, not using highways. This travel condition was defined as the common travel distance experienced by HVN nurses used in their usual day-to-day practice. The researchers developed this condition based on pre-interviews with three HVN manager nurses in A Prefecture. Meshes fully within a HVN’s reachable area were coded as ‘reachable meshes’; the population in these meshes was coded as ‘reachable population’. We calculated each municipality’s total reachable population and reachable area. Finally, we calculated each municipality’s ‘reachable proportion’ (reachable population divided by total elderly population) and ‘reachable area rate’ (reachable area divided by total area). These calculation methods were developed through discussion with four public health nursing researchers, one geographic science researcher, and three HVN managers.

We examined the proportion of home-dwelling disabled elderly people and the quality of collaboration between physicians and care managers in each municipality. The proportion of home-dwelling disabled elderly people was calculated for those aged ≥75 years and certified as care level 4 or 5; we divided the population of home-dwelling disabled elderly people by the total elderly population in each municipality. A care level of 4 or 5 indicates that the individual requires support for most activities of daily living [22]. We established the quality of collaboration between physicians and local care managers using the original prefecture public survey question ‘Do physicians and care managers in your municipality share their clients’ goals well?’ Responses used a 5-point Likert scale (1 = *not at all*; 5 = *completely*); higher scores indicated better collaboration. Public health centre officers in 13 municipalities answered this question [23].

### Individual variables

Only severely disabled individuals (Japanese long-term care insurance level 4 or 5) aged ≥75 years were included. We analysed medical and long-term care claim data from October 2012; the sample included 2641 persons who did not use hospitalization or institutional services in October 2012, and lived in 13 municipalities from which we could obtain data for all variables describing municipality characteristics. Because HVN service can be covered by medical or long-term care, in reference to clients’ physical conditions, it was crucial to analyse both types of claim data to identify the elderly people who use the HVN service. This data were limited in that only data from disabled individuals aged 75 years or older were analysed.

Data were collected describing participants’ use of HVN services, age, sex, municipality of residence, and certified care level in October 2012. Data on the presence of conditions or impairments in October 2012 and the following six months were collected to account for these conditions or impairments’ subsequent development. Data on ten types of disease were collected according to the 10th version of the International Statistical Classification of Diseases and Related Health Problems; these were as follows: 1) cancer (C00-97), 2) cerebral vascular disorder (I60, I61, I63, I69.0, I69.1, I69.3), 3) arthropathy (M15-19), 4) fracture (S02, S12, S22, S32, S42, S52, S62, S72, S82, S92, T02, T08, T10, T12), 5) pneumonia (J12-18), 6) chronic obstructive pulmonary disease (J41-44), 7) dementia (F01, F03, G30), 8) psychiatric disorder (F20-48), 9) neurological disorder (G00-29, G31-99), and 10) ischemic heart disorder (I20-25).

All municipality and individual demographic information were collated; coefficients of correlation between municipalities’ reachable population proportion and reachable area rate were subsequently calculated. HVN usage’s bivariate relationship with individual variables was then analysed. We then fitted multilevel logistic models that included random intercepts for municipalities and individual- and municipality-level independent variables. Individual variables that were significant at *p* < 0.1 and all municipality variables were included in this model. The model fitness was assessed by calculating Akaike and Bayesian information criterion. All statistical analysis was performed using SPSS v.21.

## Results

Table 1 presents municipalities’ characteristics. Municipalities’ reachable population proportion ranged from 0.0 to 90.2%. Five municipalities’ reachable proportion was <50.0%; three scored >80.0%.

**Table 1:** Sample size and characteristics by municipality

	Basic information							
Municipality	Sample size	Elderly population	Area		Reachable population (Proportion)	Reachable area	Proportion of home-dwelling disabled elderly	Quality of coordination between physician and care manager
		(n)	(km^2^)	(number of meshes)	(n; %)	(number of meshes; area rate: %)	(%)	(score)
1	942	33,094	536.4	2656	27,162 (82.1%)	615 (23.2%)	43.2	2
2	299	8,202	251.3	2493	7,179 (87.5%)	160 (6.4%)	42.7	3
3	218	4,871	233.1	1331	3,410 (69.9%)	138 (10.4%)	47.5	2
4	281	5,913	872.4	6796	4,690 (79.2%)	205 (3.0%)	48.9	3
5	153	4,510	253.9	1911	3,645 (80.7%)	176 (9.2%)	54.2	2
6	260	7,800	84.6	325	6,333 (81.2%)	170 (52.3%)	38.3	1
7	–	4,331	117.0	1289	3,234 (74.7%)	180 (14.0%)	–	-
8	191	11,126	230.7	883	10,044 (90.3%)	406 (46.0%	47.9	4
9	–	10,890	209.7	1748	8,404 (77.4%)	392 (22.4%)	–	–
10	–	2,768	94.4	359	2,534 (91.5%)	134 (37.3%)	–	–
11	10	800	194.7	860	0 (0.0%)	0 (0.0%)	30.0	3
12	–	2,104	343.7	2446	448 (21.4%)	21 (0.9%)	-	–
13	80	3,684	153.2	1330	1,393 (37.8%)	148 (11.1%)	26.9	3
14	71	1,706	152.3	1625	1,059 (63.9%)	77 (4.7%)	25.9	2
15	–	1,614	72.4	1494	1,378 (85.1%)	99 (6.6%)	–	–
16	44	1,360	212.2	1743	8 (0.6%)	6 (0.3%)	43.2	4
17	92	2,775	178.5	1484	895 (32.3%)	79 (5.3)	47.8	4

*HVN* home visiting nurse

**Fig. 3:**
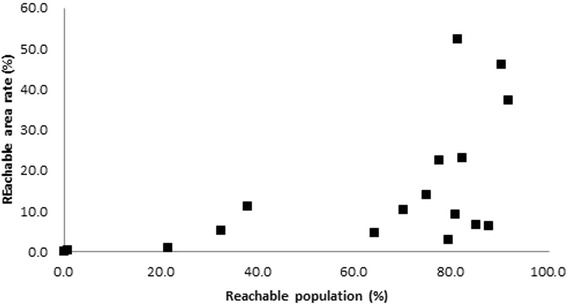
Correlation between reachable proportion (%) and reachable area rate (%) in each municipality

Figure 3 presents the correlation between reachable population proportion and reachable area rate; these variables were significantly positively correlated (Pearson’s *r* = 0.563, *p* = 0.019); however, the scatter diagram showed non-linear trends; particularly, no association was found when municipalities’ reachable population = 80.0%.

Table 2 presents statistics describing individual variables and HVN service usage. About 20% of the sample used HVN services; 33.3% were men; the sample’s average age was 87.5 years; and 59.0% had level 4 care requirements. The most common condition or impairment was cerebral vascular disorder (13.3%), followed by dementia (10.5%). Younger men with higher care levels and the presence of cancer or cerebral vascular or neurological disorders tended to use HVN significantly more.

**Table 2:** Individual characteristics and HVN service use

			Home visiting nursing service use		
		Total	Not used	Used	
		*N* = 2641	*N* = 2088	*N* = 553	*P*
Age (mean (SD)) (range)		87.5(6.9) (75–114)	87.9(7.0) (75–114)	86.0(6.1) (75–103)	<.001^b^
Sex	men	880 (33.3)	666 (75.7)	214 (24.3)	0.003^c^
	women	1761 (66.7)	1422 (80.7)	339 (19.3)	
Care level	Level 4	1559 (59.0)	1304 (83.6)	255 (16.4)	<.001^c^
	Level 5	1082 (41.0)	784 (72.5)	298 (27.5)	
Presence of conditions or impairment					
Cancer	present	123 (4.7)	87 (70.7)	36 (29.3)	0.020^c^
Cerebral vascular disorder	present	350 (13.3)	240 (68.6)	110 (31.4)	<.001^c^
Arthropathy	present	69 (2.6)	50 (72.5)	19 (27.5)	0.172^c^
Fracture	present	153 (5.8)	125 (81.7)	28 (18.3)	0.409^c^
Pneumonia	present	87 (3.3)	66 (75.9)	21 (24.1)	0.456^c^
COPD^a^	present	23 (0.9)	16 (69.6)	7 (30.4)	0.261^c^
Dementia	present	277 (10.5)	227 (81.9)	50 (18.1)	0.212^c^
Psychiatric disorder	present	109 (4.1)	92 (84.4)	17 (15.6)	0.161^c^
Neurological disorder	present	171 (6.5)	109 (63.7)	62 (36.3)	<.001^c^
Ischemic heart disease	present	101 (3.8)	75 (74.3)	26 (25.7)	0.23^c^

^a^COPD: Chronic obstructive pulmonary disease

^b^Independent *t*-test

^c^Chi-squared test

Reachable proportion was significantly positively correlated with HVN use (OR: 1.938; confidence interval [CI]: 1.265–2.967; Table 3). Age, care level, presence of cancer or cerebral vascular or neurological disorder, and quality of collaboration were also significantly associated with HVN use.

**Table 3:** Multilevel logistic regression models for HVN service use

	CrudeOdds ratio (95% CI)	Adjusted Odds ratio
Individual variables		
Age (years)	0.96 (0.95–0.97)	0.97 (0.95–0.97)
Sex (women = 1, men = 0)	0.72 (0.60–0.88)	0.89 (0.72–0.89)
Care level 5 (care level 5 = 1, care level 4 = 0)	2.00 (1.60–2.49)	2.05 (1.64–2.05)
Cancer (present = 1, absent = 0)	1.62 (1.21–2.17)	1.66 (1.18–1.66)
Cerebral vascular disorder (present = 1, absent = 0)	1.91 (1.60–2.29)	1.72 (1.41–1.72)
Neurological disorder (present = 1, absent = 0)	2.30 (1.88–2.29)	2.04 (1.52–2.04)
Municipality variables		
Reachable proportion	1.17 (0.57–2.40)	1.94 (1.27–1.94)
Quality of coordination between physicians and care managers	1.33 (1.15–1.53)	1.40 (1.23–1.60)
Number of elderly population	1.00 (0.99–1.00)	1.00 (0.99–1.01)
Proportion of home-dwelling disabled population	1.01 (0.99–1.04)	1.00 (0.97–1.03)
Constant term		
Model fitness		
Akaike information criterion	12637.75	
Bayesian information criterion	12643.62	

*HVN* home visiting nurse

Crude Odds ratio was calculated in univariate analysis with multilevel analysis

## Discussion

This study examined the relationship between HVN service use and the proportion of elderly people living within reach of HVN agencies. Municipalities with a higher reachable proportion of elderly residents showed significantly higher HVN service use rates after adjusting for individual variables; residents living in municipalities with a smaller reachable proportion are therefore more likely to have unmet HVN service needs. Public health interventions aiming to increase HVN service use should therefore increase the reachable proportion of municipalities’ elderly population through targeted service allocation.

Municipalities’ reachable area ranged from 0.0 to 52.3%; their reachable population ranged from 0.0 to 90.2%. These two variables were naturally positively correlated; however, the trend was not linear, particularly when the reachable population exceeded 80.0%. Two types of municipalities had high reachable population: those whose area was mostly within reach of HVN service agencies (i.e., high rate of reachable area), and those with a lower rate of reachable area but with densely localized and populated areas efficiently covered by HVN agencies. In urban municipalities containing only areas of high population density, reachable area could be used as a proxy for reachable population; however, this was unfeasible in rural areas because population density varied dramatically between areas.

Multilevel analysis of HVN service use indicated that elderly people who lived in municipalities with a higher reachable proportion were more likely to use HVN services. This result indicates the effect size of agencies’ geographical distribution on service use, supporting Andersen & Newman [4]. This association may inform use of home care service allocation management, as follows. Municipalities’ reachable population proportion may usefully indicate elderly populations’ access to HVN agencies. Historically, accessible service volume per person has been estimated using municipalities’ accessible service volume divided by the size of the target population [24]; however, municipalities’ reachable population proportion may be a better index of accessibility as people may use HVN services from any agency in any municipality. This accessibility index is calculated in a simple way, making it more understandable for non-professional stakeholders in long-term care system planning. Public health professionals can also use municipalities’ reachable population proportion as an index of municipality system assessment results to improve their planning. They can extend HVNs’ reachable areas with additional HVN agencies or by improving the traffic environment to maximize reachable proportions and HVN usage.

The present regression analysis’ results may explain HVN service usage rates. Current theoretical models indicate that increasing distance from peoples’ residence to health care resources delays service usage: people who face higher transportation costs for health care resources are less likely to use a give service [25]. However, travel time for HVN service usage affects HVN nurses, rather than their clients. In order to maximize the proportion of nurses’ working time spent in clients’ homes, HVN agencies may preferentially service elderly people living near the agency [26]. This would make it more difficult for elderly people farther from HVN agencies to use HVN services. Analysing service agencies’ transportation costs and nurses’ working time efficiency may help to explain the association between geographic distribution and use of HVN services.

This study is subject to the following limitations. Our analytical framework did not include caregiver needs or detailed medical conditions; previous research has found that these factors are related to home care service needs and use [13, 27]. Additionally, one rural prefecture and its 13 municipalities were analysed; our results therefore may not generalize to urban municipalities. Further, we did not analyse HVN service agency characteristics. Regarding transportation costs, nursing workload (a service agency characteristic) is associated with agency visit efficiency [28].

## Conclusion

Municipalities with a higher reachable proportion of elderly residents showed significantly higher HVN service use rates. Future research should identify mechanisms underlying reachable population proportion’s association with HVN service use. Measuring individuals’ distance from their nearest HVN agency and analysing individual and HVN agency variables may facilitate this aim. In addition, developing more user-friendly tools for calculating municipalities’ reachable population proportion may help public health professionals to develop and evaluate HVN agency allocation strategies. Achieving such aims would increase the HVN service system’s efficiency and effectiveness.

## Abbreviations

GIS: Geographic information system
HVN: Home visiting nurses

## References

[1] Colombo F, Llena-Nozal A, Mercier J, Tjadens F. OECD health policy studies, help wanted?: providing and paying for long-term care, OECD health policy studies. OECD Publishing; 2011. Retrieved from http://dx.doi.org/10.1787/9789264097759-en.

[2] Ministry of Health, Labor and Welfare. Zaitakuiryokaigorenkeisuishinjigyonotebikinitsuite [Guidelines for programs establishing home care medical and long-term care service collaboration]. 2015. Retrieved from http://www.zenhokan.or.jp/pdf/new/tuuti206.pdf.

[3] Miyazawa H (2003). Uneven nursing care service opportunity and the behavior of service providers under the long-term care insurance system. Geo Rev Jpn.

[4] Andersen R, Newman JF (2005). Societal and individual determinants of medical care utilization in the United States. Milbank Q.

[5] Cinnamon J, Schuurman N, Crooks VA (2008). A method to determine spatial access to specialized palliative care services using GIS. BMC Health Serv Res.

[6] Guagliardo MF (2004). Spatial accessibility of primary care: Concepts, methods and challenges. Int J Health Geographics.

[7] Todd A, Copeland A, Husband A, Kasim A, Bambra C (2015). Access all areas? An area-level analysis of accessibility to general practice and community pharmacy services in England by urbanity and social deprivation. BMJ Open.

[8] Statistics and Information Department, Ministry of Health, Labor and Welfare. Kaigokyufuhi geppo, 4gatsushinsabun, 2014 [Survey of Long-term Care Benefit Expenditures, monthly report (April, 2014)]. Retrieved from http://www.mhlw.go.jp/toukei/list/45-1b.html

[9] Markle-Reid M, Browne G, Weir R, Gafni A, Roberts J, Henderson SR. The effectiveness and efficiency of home-based nursing health promotion for older people: a review of the literature. Med Care Res Rev : MCRR;63(5):531–69. doi:10.1177/1077558706290941. 10.1177/1077558706290941 16954307

[10] Social Statistics Division, Japan. Kaigosabis shisetsu, jigyoushochosa, 2014 [Survey of institutions and establishments for long-term care (2014)]. Retrieved from http://www.e-stat.go.jp/SG1/estat/List.do?lid=000001117885

[11] Nagata S, Taguchi A, Naruse T, Kuwahara Y, Murashima S (2013). Unmet needs for visiting nurse services among older people after hospital discharge and related factors in Japan: cross-sectional survey. Jpn J Nurs Sci.

[12] Naruse T, Taguchi A, Nagata S, Kuwahara Y, Murashima S (2012). Prevalence of home visiting nurse service clients who received insuffi cient number of nurse visits in the Japanese long-term care insurance. Japanese J Nurs Heal Sci.

[13] Naruse T, Nagata S, Taguchi A, Murashima S (2010). Classification tree model identifies home-based service needs of Japanese long-term care insurance consumers. Pub Health Nurs.

[14] Asai MO, Kameoka VA (2005). The influence of Sekentei on family caregiving and underutilization of social services among Japanese caregivers. Soc Work.

[15] Cho SH (2005). Older people’s willingness to use home care nursing services. J Adv Nurs.

[16] Forbes DA, Stewart N, Morgan D, Anderson M, Parent K, Janzen BL (2003). Individual determinants of home-care nursing and housework assistance. Can J Nurs Res.

[17] Kim EY, Cho E, June KJ (2006). Factors influencing use of home care and nursing homes. J Adv Nurs.

[18] Li H (2006). Involvement of informal and formal service providers: meeting the home care needs of older adults with severe functional impairments. Home Health Care Serv Q.

[19] Goyal NK, Hall ES, Jones DE, Meinzen-Derr JK, Short JA, Ammerman RT, Van Ginkel JB (2014). Association of maternal and community factors with enrollment in home visiting among at-risk, first-time mothers. Am J Pub Health.

[20] McGuigan WM, Katzev AR, Pratt CC (2003). Multi-level determinants of retention in a home-visiting child abuse prevention program. Child Abuse Negl.

[21] Ministry of Internal Affairs and Communication. Heisei-22-nendo Kokuseichosa [National population survey, 2010, in Japan]. 2010. Retrieved from http://www.stat.go.jp/data/kokusei/2010/.

[22] Shimizuya S. The future of long-term care in Japan. Tokyo, Japan, 2013. Retrieved from http://www.rieti.go.jp/jp/publications/summary/13070009.html.

[23] Nagata S. Reseputodetawokatsuyoshita ryoyoubashoikouto saabisuriyonotsuisekichousanimotodsuku koukatekinachiikirenkeitaiseinomeikakuka [Exploring effective within community collaboration system by using retrospective cohort survey]. Tokyo: Satoko Nagata; 2015.

[24] Ito A, Terasaki H, Hisashi D (2014). Shinryojono todofukenbunpukaramitajuminnozaitakuiryoenoakusesukakusanikansurukenkyu [A study on gaps in the access to home-based medical care by residents according to medical clinic distributions by prefecture]. J Jpn Soc for Healthcare Admin.

[25] Penchansky R, Thomas JW (1981). The concept of access: definition and relationship to consumer satisfaction. Med Care.

[26] O’Brien-Pallas L, Baumann A (2000). Toward evidence-based policy decisions: a case study of nursing health human resources in Ontario, Canada. Nurs Inq.

[27] Bass DM, Noelker LS (1987). The influence of family caregivers on elder’s use of in-home services: an expanded conceptual framework. J Health Soc Behav.

[28] Kuwahara Y, Nagata S, Taguchi A, Naruse T, Kawaguchi H, Murashima S (2013). Measuring the efficiencies of visiting nurse service agencies using data envelopment analysis. Health Care Manage Sci.

